# Identification of Microbial Species and Analysis of Antimicrobial Resistance Patterns in Acute Cholangitis Patients with Malignant and Benign Biliary Obstructions: A Comparative Study

**DOI:** 10.3390/medicina59040721

**Published:** 2023-04-06

**Authors:** Bogdan Miuțescu, Deiana Vuletici, Călin Burciu, Adina Turcu-Stiolica, Felix Bende, Iulia Rațiu, Tudor Moga, Omar Sabuni, Adnan Anjary, Sami Dalati, Bogdan Silviu Ungureanu, Eyad Gadour, Florin George Horhat, Alina Popescu

**Affiliations:** 1 Department of Gastroenterology and Hepatology, “Victor Babes” University of Medicine and Pharmacy Timisoara, Eftimie Murgu Square 2, 300041 Timisoara, Romania; 2 Advanced Regional Research Center in Gastroenterology and Hepatology, “Victor Babes” University of Medicine and Pharmacy, 30041 Timisoara, Romania; 3 Department of Pharmacoeconomics, University of Medicine and Pharmacy of Craiova, 200349 Craiova, Romania; 4 Faculty of General Medicine, Altinbas University, Dilmenler Cd., 34217 Istanbul, Turkey; 5 Faculty of General Medicine, Yeditepe University, Kayısdagı Cd., 34755 Istanbul, Turkey; 6 Faculty of General Medicine, Baskent University, Fatih Sultan, 06790 Ankara, Turkey; 7 Research Center of Gastroenterology and Hepatology, University of Medicine and Pharmacy of Craiova, 200349 Craiova, Romania; 8 Department of Gastroenterology, King Abdulaziz Hospital-National Guard Health Affairs, Al Ahsa 31982, Saudi Arabia; 9 Department of Medicine, Zamzam University College, Khartoum 11113, Sudan; 10 Multidisciplinary Research Center on Antimicrobial Resistance (MULTI-REZ), Microbiology Department, “Victor Babes” University of Medicine and Pharmacy, 300041 Timisoara, Romania

**Keywords:** acute cholangitis, antibiotic resistance, antimicrobial resistance, bile duct obstruction

## Abstract

*Background and Objectives*: Acute cholangitis (AC) is still lethal if not treated promptly and effectively. Biliary drainage, also known as source control, has been acknowledged as the backbone treatment for patients with AC; nonetheless, antimicrobial therapy allows these patients to undergo non-emergent drainage procedures. This retrospective study aims to observe the bacterial species involved in AC and analyze the antimicrobial resistance patterns. *Materials and Methods*: Data were collected for four years, comparing patients with benign and malignant bile duct obstruction as an etiology for AC. A total of 262 patients were included in the study, with 124 cases of malignant obstruction and 138 cases of benign obstruction. *Results*: Positive bile culture was obtained in 192 (73.3%) patients with AC, with a higher rate among the benign group compared with malignant etiologies (55.7%.vs 44.3%). There was no significant difference between the Tokyo severity scores in the two study groups, identifying 34.7% cases of malignant obstruction with Tokyo Grade 1 (TG1) and 43.5% cases of TG1 among patients with benign obstruction. Similarly, there were no significant differences between the number of bacteria types identified in bile, most of them being monobacterial infections (19% in the TG1 group, 17% in the TG2 group, and 10% in the TG3 group). The most commonly identified microorganism in blood and bile cultures among both study groups was *E. coli* (46.7%), followed by *Klebsiella* spp. (36.0%) and *Pseudomonas* spp. (8.0%). Regarding antimicrobial resistance, it was observed that significantly more patients with malignant bile duct obstruction had a higher percentage of bacterial resistance for cefepime (33.3% vs. 11.7%, *p*-value = 0.0003), ceftazidime (36.5% vs. 14.5%, *p*-value = 0.0006), meropenem (15.4% vs. 3.6%, *p*-value = 0.0047), and imipenem (20.2% vs. 2.6%, *p*-value < 0.0001). *Conclusions*: The positive rate of biliary cultures is higher among patients with benign biliary obstruction, while the malignant etiology correlates with increased resistance to cefepime, ceftazidime, meropenem, and imipenem.

## 1. Introduction

Acute cholangitis (AC) is a severe condition that affects the hepatobiliary system, with an associated mortality of 5–10% if treated with endoscopic biliary drainage [1,2] and approximately 50% if untreated [3]. The determining causes are diverse, with choledocholithiasis as the leading cause in 57% of cases [4], followed by malignant pathologies in 10–30% of patients [5]. The diagnosis is based on the pathognomonic signs of the dilated biliary tree, detected by diagnostic imaging [6]. The biological findings are correlated with alteration of complete blood count (CBC), increased aspartate aminotransferase (AST), alanine aminotransferase (ALT), bilirubin, gamma-glutamyl transferase (GGT), and alkaline phosphatase (ALP) [7].

The diagnosis of AC was based, until recently, on the Charcot triad, which uses as diagnostic criteria fever, abdominal pain, and jaundice [8]. However, despite its high specificity of 93–99%, its sensitivity is low (36–46%), thus making it a good confirmation test but a poor screening and diagnostic test [9,10]. All the efforts to increase the AC diagnosis were summarized in the first Tokyo Guideline from 2007 (TG07) [11], which was later updated to TG13 [6] and TG18 [12]. The Dutch Pancreatitis Study Group (DPSG) recently proposed new diagnostic criteria for AC in the presence of acute biliary pancreatitis [13].

The AC treatment is based on two management options that comprise antibiotic treatment (AT) and biliary drainage. According to TG18 [7], endoscopic retrograde cholangiopancreatography (ERCP) should be considered the first-line drainage procedure. For mild forms, the indication of drainage is correlated with the response to antibiotic treatment; however, for moderate AC, the drainage must be performed early, and for severe conditions, as soon as possible [7]. This indication is confirmed by multiple studies that concluded that early ERCP decreases the duration of hospitalization [14] and mortality [4,14]. The second management option for AC is antimicrobial treatment, where TG18 provides a large spectrum based on the three classifications of AC [15]. The importance of the administration of antibiotics has already been proven, but the duration of administration is a debated subject; some studies hypothesize that short-term AT has the same efficacy as long-term AT, with the condition that the biliary tree has been decompressed [16,17].

Cancer patients have a three-times higher risk of death from infection [18], which is a very common complication in this particular population [19]. Thus, increasing antibiotic resistance in patients with malignant diseases can lead to unfavorable outcomes after AC [20]. Although the incidence of bloodstream infection (BSI) in patients with solid tumors is lower than in hematological patients, the majority of studies have focused on patients with hematological malignancies, while the most frequent source of recurrent BSI seems to be cholangitis [21].

Multi-drug resistant (MDR) pathogens are an evolving problem in AC, especially in immunocompromised patients [22]. The factors that can lead to biliary MDR bacteria are male gender, nosocomial AC, prior antibiotic exposure, and prior biliary stenting; even so, the survival rate and hospital stay in AC patients with and without detected biliary MDR pathogens are similar [23]. Thus, in the current era of increasing antibiotic resistance, biliary cultures (BCs) are imperious, allowing for the proper adjustment of antibiotic treatment based on the antibiogram results. The positive rate of BC is directly correlated with the form of AC (80.4 vs. 82.2% vs. 88.6% in mild, moderate, and severe conditions, respectively [15], or, depending on the inclusion criteria, averaging 91.8% [24]). Studies have described that the positive rate of blood culture among patients with collected BC varies between 30% and 40%, where 87% of patients grew the same organism as their bile culture [24,25,26].

Although some studies have evaluated the microbiology of bile aspirates in patients with AC [27] and others have compared cholangitis patients with and without plastic biliary stents [28], there is still very limited information regarding bile culture and antibiotic susceptibility patterns in the malignant and benign etiologies of AC. Thus, one of the hypotheses raised by the current study assumes that there is a significant difference in the microbial species distribution between patients with AC caused by malignant biliary obstructions and those with benign biliary obstructions. Another hypothesis is that antimicrobial resistance patterns differ significantly between the microbial species isolated from patients with malignant biliary obstructions and those with benign biliary obstructions. Therefore, this study’s primary purpose is to identify and compare the microbial species present in the bile aspirates of patients with acute cholangitis (AC) associated with malignant and benign biliary obstructions; to evaluate and compare the antimicrobial resistance patterns in the isolated microbial species from the bile aspirates of patients with malignant and benign biliary obstructions; and to provide evidence-based recommendations for the empirical antibiotic treatment of AC in patients with malignant and benign biliary obstructions.

## 2. Materials and Methods

### 2.1. Study Design and Ethics

A retrospective study was performed at the Emergency County Hospital Timisoara, a tertiary care center in Western Romania. All patients who underwent an ERCP for biliary drainage due to AC between June 2018 and June 2020 were included. All patients had a bile culture sample and a blood culture sample collected. Patients’ medical data and personal information were collected from the medical records and patient files. The resistance of the bacteria to the antibiotics recommended by TG18 [15] was evaluated, and we attempted to identify the difference in antimicrobial resistance between malignant and benign bile duct obstruction causing AC. The study protocol conformed to the ethical guidelines of the 1975 Declaration of Helsinki. The internal review board approved it on 14 October 2022 (approval number I-27098).

### 2.2. Patients and Sampling

The diagnosis of AC was established using TG18 criteria 13. Patients were included in this study only once, on their first admission, despite some having more than one episode of AC during the data collection period. The exclusion criteria were: cholangitis secondary to ERCP, post-ERCP perforation, percutaneous or surgical drainage, or if the patient used antibiotic therapy (AT) for any other diseases when the AC was diagnosed.

All the patients received antibiotics according to their corresponding grade from TG18 recommendations [15] after admission, and the diagnosis of AC was established. In the department where the study was performed, the most common antibiotic schemes used for mild AC were ampicillin/sulbactam, ciprofloxacin, or levofloxacin for mild AC; ceftriaxone, cefepime, or piperacillin-tazobactam for moderate AC; and meropenem or imipenem for severe AC. Microorganisms from blood and bile samples were identified on culture media. The blood culture samples were collected at admission for the patients with moderate and severe forms of AC, according to TG18’s recommendation. Because of our center’s particularity as a tertiary endoscopy department, where patients are referred from different healthcare facilities, not all had a blood culture collected before initiating antibiotic therapy. Bile samples were obtained after cannulation via the sphincterotome before the therapeutic procedure. Firstly, at least 5 mL of bile that was collected was discarded; immediately after that, another 5 mL of bile was collected in a sterile tube containing a medium for anaerobic, aerobic bacterial cultures. The samples were incubated for at least seven days at 37 °C until microbial growth was detected. Antibiotic susceptibility testing (MIC) was performed using a VITEK^®^ 2 system (bioMérieux, Marcy-l′Étoile, France) with the results interpreted according to the existing guidelines [29]. Clinical and Laboratory Standards Institute (CLSI) recommendations and criteria for all bacteria cultured were used to define susceptibility to antimicrobial agents [30].

On admission, B-mode ultrasonography was used to determine the cause of the obstruction. If a diagnosis could not be made, we performed an endoscopic ultrasound (EUS) procedure, contrast-enhanced computer tomography (CT), or CE magnetic resonance imaging (MRI), which are also used in the staging of malignant causes. In addition, we examined the tumor markers and histopathological findings from the ERCP or EUS biopsies to confirm the diagnosis. ERCP was used only as a therapeutic tool, performed with a therapeutic duodenoscope (Olympus Corp., Tokyo, Japan), and common bile duct cannulation was done using a guidewire. All ERCP procedures were performed under sedation using midazolam, propofol, and fentanyl by a team from anesthesia and intensive care; the drugs were combined according to their internal protocols. The timing of ERCP was established according to the severity of the disease and Tokyo Guidelines criteria by experienced endoscopists. The main goal of the ERCP for patients with choledocholithiasis was extracting the stones. In cases of complicated choledocholithiasis, which is difficult to extract, we placed plastic stents. For other cases, we placed plastic or metal stents, depending on the diagnosis.

### 2.3. Data Collection and Variables

Demographic data and the patient’s medical history were collected from the patient’s discharge reports. The variables considered for analysis comprised demographic data: the etiology of infection, the clinical characteristics of the study population (age, gender, age category, signs and symptoms, presence of bile duct stents, history of cholecystectomy, ERCP timing, hospitalization, Tokyo severity score), bacterial identification in bile (Gram-positive and Gram-negative organisms), and antimicrobial resistance patterns. The study included four antibiotics classes: penicillin, cephalosporins, fluoroquinolones, and carbapenems. These antibiotics were tested by the hospital per laboratory guidelines and are recommended by the Tokyo Guidelines 2018 for the treatment of AC until bile or blood culture validation.

### 2.4. Statistical Analysis

GraphPad Prism v9.2.0. (GraphPad, San Diego, CA, USA) and R statistical software version 4.0.3 (2021, GNU General Public License) were used for the statistical analysis. Continuous variables were given as mean (standard deviation) or median (interquartile range), while categorical variables were expressed as the number of subjects (*n*) and the percentage value (%). The distribution of continuous variables was tested by the D’Agostino–Pearson omnibus normality test, revealing the data to be nonparametrically distributed. Hence, nonparametric two-way analysis was performed using the Mann–Whitney U-test. Radar plots were designed using the Plotly package to distinguish the multidimensional data of antibiograms. Fisher’s exact or chi-square test, two-sided, was used to compare categorical variables. The results were considered statistically significant, with a *p*-value of <0.05.

## 3. Results

### 3.1. Clinical Characteristics of the Study Population

A total number of 262 patients were included in this study. The etiology of cholangitis was analyzed in Table 1. Most patients in the benign group were diagnosed with choledochal lithiasis (48.5%). Forty-seven percent of patients (*n* = 124/262) were diagnosed with a malignant pathology, the leading cause being pancreatic cancer (24.8%). The mean age between the two groups had no statistical differences (*p*-value = 0.93); however, patients from the middle age category were more frequent in the malignant group and young patients in the benign group. No differences were found in gender between malignant and benign patients (*p*-value = 0.508), as seen in Table 2. Abdominal pain was more frequent in the benign group compared to the malignant group (80.4% vs. 58.1%, *p*-value < 0.0001). In patients presenting fever, no differences were observed between the two groups (*p*-value = 0.187). Prolonged hospital stay was more frequently associated with malignant diseases than benign ones (*p*-value = 0.04).

### 3.2. Bacterial Identification

According to the Tokyo Guidelines of 2018, monomicrobial growth was found more in patients with mild severity (19%), followed by moderate severity (17%) and the severe grade (10%), as seen in Table 3. Monomicrobial growth was the most encountered (46%) in comparison with sterile (26%) or polymicrobial cultures (two bacteria—24% or three bacteria—3%). Cultures were positive in 192 of 262 bile specimens (73.3%), most of them (107/192, 55.7%) having a benign etiology for the acute obstruction of the main biliary duct; 44.3% of patients (85/192) had a malignant etiology of acute cholangitis.

Table 4 describes a detailed comparison of isolated microorganisms from blood cultures and bile cultures between patients with malignant and benign obstruction causes. The most frequently encountered in bile cultures were Gram-negative bacteria, including *Escherichia coli* (*E. coli*) (37.6% for patients with malignant disease vs. 56.1% for patients with benign disease, *p* = 0.003), *Klebsiella* (29.4% for patients with malignant disease vs. 24.3% for patients with benign disease, *p* = 0.876), *Pseudomonas* (14.1% for patients with malignant disease vs. 10.3% for patients with benign disease, *p* = 0.667), and *Citrobacter* (7.1% for patients with malignant disease vs. 4.7% for patients with benign disease, *p* = 0.760). The most frequently encountered Gram-positive bacteria was *Enterococcus* (24.7% for patients with malignant disease vs. 18.7% for patients with benign disease, *p* = 0.612).

Of 262 patients with AC, 141 (53.8%) had a collected blood culture and 97 (68.8%) were sterile; from this amount, 67 (69%) had a positive bile culture. Bacterial growth in the blood culture was found in 31% of the patients. Of 44 patients with positive hemoculture, there was one bacterium grown in 41 (29%) patients and two bacteria grown in 3 (2%) patients. Of positive blood cultures, 29 (65%) had a similar germ with the bile culture, and 4 (9%) had negative bile culture. It was observed that the most frequent organism identified in blood cultures was *E. coli*, with 31.3% of all malignant obstructions and 55.2% in benign obstructions, respectively, and similarly in bile cultures, 50.0% in malignant cases vs. 40.9% in benign cases. The next-in-frequency organisms identified were Klebsiella spp. in approximately 20% of all blood cultures and Pseudomonas in about 5%. No statistical differences of isolated microorganisms were found in blood cultures comparing patients with malignant and benign diseases, as shown in Table 5.

### 3.3. Antibiogram Study

**Figure 1:**
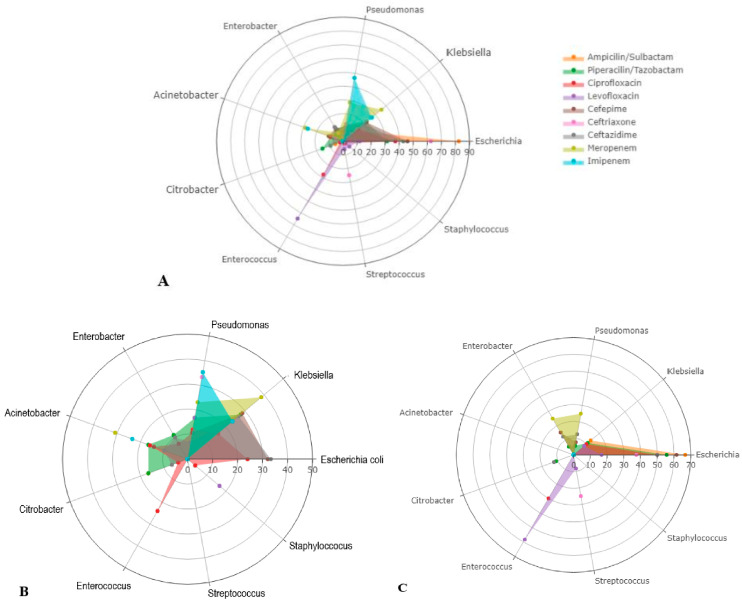
Radar plots reporting resistance microbial sensitivity of given antibiotics for patients with acute cholangitis. All patients (**A**), malignant (**B**), benign (**C**). Figures generated with Plotly data analytics and visualization tools.

A total of 266 antibiograms were analyzed, with 119 from patients with malignant obstruction and 147 from those with benign obstruction. The table summarizes the percentage of antibiotic resistance for each antibiotic in both groups, along with the statistical significance of the differences observed. Ampicillin/sulbactam showed resistance in 31.3% of the total cases (21/67), with 27.2% (6/22) resistance in malignant cases and 33.3% (15/45) in benign cases. The difference in resistance between the two groups was not statistically significant (*p* = 0.780). Piperacillin/tazobactam resistance was present in 17.3% of the total cases (36/207), with 19.7% (18/91) resistance in malignant cases and 15.6% (18/115) in benign cases. The difference was also not statistically significant (*p* = 0.464). According to the data from Table 6 and Figure 1, cefepime (*p*-value = 0.001), ceftazidime (*p*-value = 0.001), meropenem (*p*-value = 0.004), and imipenem (*p*-value < 0.001) had significantly higher resistance rates for the malignant group of patients than for the benign group. There were no significant differences in antibiotic resistance between the two groups for the other evaluated antibiotics recommended by the TG18.

A detailed breakdown of the bacterial resistance to antibiotics from bile culture is presented in Table 7, where the highest resistance profile was observed in samples positive with *Escherichia coli.* We observed a high resistance pattern of *E. coli* to ampicillin/sulbactam in benign (46.2%) and malignant (36.4%) cases. The second highest antimicrobial resistance of *E. coli* was identified for ceftazidime (33.3% in malignant cases and 22.6% in benign cases) and cefepime (31.0% in malignant cases and 16.7% in benign cases), respectively. The second most commonly identified bacteria was *Klebsiella* spp. in 27.1% of patients, having a high antimicrobial resistance pattern to ampicillin/sulbactam (33.3%) and ceftriaxone (22.2%) in patients with benign obstructions. *Klebsiella* spp. was also highly resistant to cefepime in malignant cases (34.8%) and piperacillin/tazobactam in 18.5% of benign cases, respectively. The third most commonly involved bacteria was *Enterococcus* spp. in 21.4% of patients, equally between the malignant and benign causes of obstruction. *Enterococcus* was resistant to ciprofloxacin in 50% of benign cases and 40% of malignant cases and to levofloxacin in 50% of benign cases and 26.7% of malignant patients.

## 4. Discussion

### 4.1. Current Findings and Published Data

Based on the findings, the current study carries significant implications for the clinical management of acute cholangitis patients with malignant and benign biliary obstructions by providing valuable insights into the microbial species and their antimicrobial resistance patterns in these patient groups. Among the study’s key findings, it was observed that *E. coli* was the most frequently encountered bacteria in bile cultures, with a higher prevalence in patients with benign disease than malignant disease (56.1% vs. 37.6%, *p* = 0.003). Moreover, cefepime, ceftazidime, meropenem, and imipenem had significantly higher resistance rates in the malignant group compared to the benign group (*p*-values < 0.05). These results are important because they can help guide appropriate empirical antibiotic therapy selection for acute cholangitis patients based on their underlying biliary obstruction etiology. For instance, the higher resistance rates observed for certain antibiotics in the malignant group may warrant the use of alternative antibiotics in these patients. Furthermore, identifying the most frequently encountered microorganisms in bile cultures, such as *E. coli*, can help develop targeted therapies.

The choice of antimicrobial treatment depends on the severity of cholangitis and local resistance to antibiotics. Therefore, updates for local antibiograms are necessary to provide efficient therapy in clinical practice. Previously published data show a positive rate of bile cultures for patients with AC, ranging from 28% to 93% [27,31]. In the present study, a positive bile culture was obtained in 192 patients with AC, representing 73.3% of the entire cohort, with a higher rate among the benign group than the malignant one (55.7% vs. 44.3%). On the other hand, 69% of blood cultures were sterile, compared with only 26.7% of sterile bile cultures. It was previously observed in other studies that positive rates of blood cultures among patients with AC range from 21% to 71% [32].

Our study confirmed the benefit of bile culture over blood culture since the positive rate was much higher in BC compared with blood culture. However, in the cases of positive blood culture, the same germ in BC was found in 65% of cases, lower than the data presented by Chandra [26]. The superiority of BC was proven by a lower number of false-negative bile cultures compared with false-negative blood cultures. The high sensitivity and low specificity of TG18, as opposed to Charco’s triad, which has low sensitivity but very high specificity, can explain the number of false-negative AC cases [10]. Recent data published by Gromski et al., where the AC diagnostic criteria were similar to Charcot’s triad, showed a positive BC rate of 91.8% [24], higher than our study’s rate.

In this study, *E. coli* was the predominant isolate in both groups. However, it was statistically more present in patients with benign disease (37.6% vs. 56.1%, *p*-value = 0.003). *Klebsiella* spp. was the second most commonly identified germ, which was more frequent in malignant etiologies (29.4% vs. 24.3%). The main findings of the present study are consistent with the Study for Monitoring Antimicrobial Resistance Trends (SMART) results, which reported culture and antimicrobial susceptibility data from intra-abdominal collections. Gomi et al. published a large-cohort multicenter observational study in 2017 among patients with AC, where the most frequent organism found in bile culture was *Escherichia coli* [33]. Similarly, review studies reported that coliform organisms such as *Escherichia coli* (25–50%), *Klebsiella* spp. (15–20%), and *Enterobacter* species (5–10%) are among the most commonly identified bacteria [34,35], while Enterococcus species were identified in 10–20% of infections. Occasionally, anaerobic bacteria such as *Bacteroides fragilis* and *Clostridium perfringens* may also induce AC, especially in individuals with a history of biliary operations and the geriatric population. However, these pathogens were not identified in our research, likely due to insufficient sample size or due to the high number of false-negative results that are often found in anaerobic bacteria [36,37].

The Tokyo Guidelines suggest using beta-lactamase or cephalosporine-based antimicrobial therapy in mild cholangitis. In our study, the resistance to ampicillin/sulbactam was above 20%, consistent with the information found in the Tokyo Guidelines, without significant differences between malignant and benign etiologies of cholangitis (22.2% vs. 33.3%). It was observed that the overall increased resistance rate of *E. coli* has an increasing trend for ampicillin/sulbactam [38]. Similar findings were seen in our study, where resistance was higher in benign cases than in malignant cases. These findings prompted us to change the current antibiotic therapy protocol so that empirical ampicillin/sulbactam treatment is no longer given in AC, except in cases where a previous bile culture was sensitive to this antibiotic. The treatment will then be modified based on the results of the bile culture from the current hospitalization.

In addition, in benign cases, *Klebsiella* spp. susceptibility to ampicillin/sulbactam was reduced. This could be due to the extensive use of this antibiotic in the absence of studies demonstrating local susceptibility to ampicillin/sulbactam. Furthermore, the proportion of mild forms of benign AC is greater than the proportion of malignant AC; in this case, over-exposure to ampicillin/sulbactam can lead to decreased susceptibility. For fluoroquinolones, also recommended in mild forms of AC, the resistance is around 20%, without differences between etiologies. For moderate cholangitis, TG18 suggests using cephalosporine; in our study, the resistance to ceftriaxone was similar in both etiologies.

However, we found that patients with cancer have greater levels of cefepime and ceftazidime resistance. This discrepancy can be attributed to the increasing resistance of *E. coli* and *Klebsiella* spp., the two most prevalent germs identified in patients with cancer in our study. *Klebsiella* spp. is part of the ESKAPE group, commonly associated with antibiotic resistance in hospital settings. Knowing the increased risk of infection in cancer patients, a possible causality can be linked to the intensive use of this antibiotic in other infections [39]. However, the possibility of other confounding factors cannot be ruled out. Meropenem and imipenem, prescribed for severe types of AC, have shown higher resistance in malignant patients compared to benign cases (15.4% vs. 3.6% and 20.2% vs. 2.6%, respectively). These findings can be explained by the significant carbapenem resistance of *Klebsiella* spp. in malignant patients due to biofilm production, as described by other studies [40]. The molecular mechanisms of carbapenem resistance in *Enterobacteriaceae* are represented by two major mechanisms: β-lactamase activity combined with structural mutations and the production of carbapenemases, enzymes that hydrolyze carbapenem antibiotics [41]. Carbapenemase-producing *Klebsiella pneumoniae* isolated from pediatric cancer patients has been reported [42]. Other studies have reported the capability of *Klebsiella* spp. to acquire resistance to fourth-generation cephalosporins, information that can be correlated with our findings in antimicrobial resistance [43]. However, the resistance and susceptibility of *Klebsiella* spp. to sulbactam and associated combinations remain a debate [44,45].

### 4.2. Study Strengths and Limitations

As a retrospective and descriptive study, doubts can be raised about the extent of the result’s applicability to other settings. This study was not intended to be a final work but, rather, only an initial work documenting the antimicrobial susceptibilities of biliary pathogens. At this time, the study is best when limited to the area where the study facility is located and to the population of the country. Being a tertiary hospital that assures ERCP for four counties, not all patients had blood cultures collected; therefore, selection bias can occur. Other biases can happen due to missing data. Another limitation is that not all the antibiotics evaluated had been tested for all germs, a condition generated by the study’s retrospective design. In the future, we need prospective studies that will test TG-recommended antibiotics for all germs and can validate the results of this study. Aside from the limitations mentioned above, the current study adds to the existing literature by providing new evidence on bacterial identification in biliary tract infections and the spectrum of antimicrobial resistance in the context of alarmingly increasing resistant infections, particularly in malignant patients. Nevertheless, based on our findings, future studies can explore in-depth specific classes of antibiotics with a potential application in treating AC as well as include the detection of beta-lactamase and carbapenemase in the tested samples. Because very few studies on acute cholangitis have been published with the same goals, more multicentric studies are needed to gain a better understanding of the bacteria involved and the spectrum of antimicrobial resistance.

## 5. Conclusions

In conclusion, the positive rate of biliary culture was higher in patients with acute cholangitis of a benign cause. Overall, patients with malignant causes of obstruction showed a higher rate of antimicrobial resistance and exhibited a different spectrum of pathogens. Our findings could help in establishing empiric antibiotic therapy in complicated cases of AC, providing a higher success rate of the empiric treatment. A multidisciplinary approach might be beneficial to the discussion and provision of appropriate antimicrobial agents in the institution, region, and country.

## Figures and Tables

**Table 1:** Comparison of etiology between patients with malignant and benign disease.

Etiology	Total (*n* = 262)
Benign	Total 138 (52.7%)
Choledocholithiasis	127 (48.5%)
Vaterian ampulloma	5 (1.9%)
Benign coledochal stenosis	3 (1.1%)
Mirizzi syndrome	2 (0.8%)
Liver abscess	1 (0.4%)
Malignant	Total 124 (47.3%)
Pancreatic cancer	65 (24.8%)
Cholangiocarcinoma	35 (13.4%)
Malignant vaterian ampulloma	14 (5.3%)
Malignant extrinsic compression	7 (2.7%)
Gallbladder cancer	3 (1.1%)

**Table 2:** Clinical characteristics of the study population stratified by etiology of biliary obstruction.

Variables	Total (*n* = 262)	Malignant (*n* = 124)	Benign (*n* = 138)	Significance
Gender (male)	128 (48.9%)	61 (49.2%)	67 (48.6)	0.508
Age, mean (SD)	67.6 (14.1)	68.5 (11.3)	66.8 (16.2)	0.330
Age, median (IQR)	70.0 (19.0)	69.5 (16.8)	70.0 (22.2)	0.930
Age category				0.005
Young adults (18–39 years)	15 (5.7%)	2 (1.6%)	11 (8.0%)	
Middle age (40–65 years)	84 (32.1%)	50 (40.3%)	34 (24.6%)	
Older adults (>65 years)	159 (60.7%)	72 (58.1%)	87 (63.0%)	
Abdominal pain, yes	183 (69.8)	72 (58.1%)	111 (80.4%)	<0.001
Jaundice	234 (89.3%)	117 (94.4%)	117 (84.8%)	0.015
Fever	85 (32.4%)	35 (28.2%)	50 (36.2%)	0.187
Previous stent, yes	48 (18.3%)	41 (33.1%)	7 (5.1%)	<0.001
Cholecystectomy, yes	50 (19.1%)	19 (15.3%)	31 (22.5%)	0.158
ERCP timing				0.912
Emergent (<48 h)	176 (67.2%)	83 (66.9%)	93 (67.4%)	
Urgent (48–72 h)	44 (16.8%)	20 (16.1%)	24 (17.4%)	
Late (>72 h)	42 (16%)	21 (16.9%)	21 (15.2%)	
Hospitalization days	7 (4–10)	7 (5–10)	6 (4–10)	0.040
Weekend admission, yes	73 (27.9%)	29 (23.4%)	44 (31.9%)	0.132
Tokyo severity score,				0.075
Grade I	103 (39.3%)	43 (34.7%)	60 (43.5%)	
Grade II	95 (36.3%)	43 (34.7%)	52 (37.7%)	
Grade III	64 (24.4%)	38 (30.6%)	26 (18.8%)	

Data reported as *n* (%) and calculated using the chi-square test and Fisher’s exact test unless specified differently; mean and SD values compared with Student’s t-test; median and IQR values compared with Mann–Whitney U-test; IQR—interquartile range; SD—standard deviation.

**Table 3:** Bacterial presence in bile according to Tokyo Guidelines.

Tokyo Grade	Grade I (*n* = 103)	Grade II (*n* = 95)	Grade III (*n* = 64)	Significance
Sterile	26 (10%)	27 (10%)	17 (6%)	0.973
1 bacterium	50 (19%)	44 (17%)	27 (10%)	
2 bacteria	24 (9%)	21 (8%)	17 (6%)	
3 bacteria	3 (1%)	3 (1%)	3 (1%)	

Proportions evaluated with a chi-square test.

**Table 4:** Comparison of isolated microorganisms from bile cultures between patients with malignant and benign etiologies of obstruction.

Isolated Microorganisms from Bile Cultures No. of Patients (%)	Total (*n* = 192)	Malignant (*n* = 85)	Benign (*n* = 107)	Significance
Gram-negative organisms				
*Escherichia coli*	92/192 (47.9%)	32 (37.6%)	60 (56.1%)	0.003
*Klebsiella* spp.	51/192 (26.6%)	25 (29.4%)	26 (24.3%)	0.876
*Pseudomonas* spp.	25/192 (13%)	12 (14.1%)	11 (10.3%)	0.667
*Enterobacter* spp.	10/192 (5.2%)	4 (4.7%)	6 (5.6%)	0.752
*Acinetobacter* spp.	6/192 (3.1%)	5 (5.9%)	1 (0.9%)	0.104
*Citrobacter* spp.	11/192 (5.7%)	6 (7.1%)	5 (4.7%)	0.760
Gram-positive organisms				
*Enterococcus* spp.	41/192 (21.6%)	21 (24.7%)	20 (18.7%)	0.612
*Streptococcus* spp.	6/192 (3.1%)	1 (1.2%)	5 (4.8%)	0.217
*Staphylococcus* spp.	3/192 (1.6%)	2 (2.4%)	1 (0.9%)	0.604

Data reported as *n* (%) and calculated using the chi-square test and Fisher’s exact test unless specified differently.

**Table 5:** Comparison of isolated microorganisms from blood cultures between patients with malignant and benign causes of obstruction.

Isolated Microorganisms from Blood Cultures, *n* (%)	Malignant Disease (*n* = 16/127)	Benign Disease (*n* = 29/138)	Significance
Sterile	109 (85.8%)	111 (80.4%)	0.242
Gram-negative organisms			
*Escherichia coli*	5 (31.3%)	16 (55.2%)	0.123
*Klebsiella* spp.	4 (25.0%)	5 (17.2%)	0.533
*Pseudomonas* spp.	1 (6.3%)	1 (3.4%)	0.662
*Enterobacter* spp.	1 (6.3%)	3 (10.3%)	0.644
*Acinetobacter* spp.	1 (6.3%)	0 (0.0%)	0.173
*Citrobacter* spp.	0 (0.0%)	1 (3.4%)	0.452
Gram-positive organisms			
*Enterococcus* spp.	1 (6.3%)	1 (3.4%)	0.662
*Staphylococcus* spp.	3 (18.8%)	1 (3.4%)	0.084
*Streptococcus* spp.	0 (0.0%)	1 (3.4%)	0.452

Data reported as *n* (%) and calculated using the chi-square test and Fisher’s exact test unless specified differently.

**Table 6:** Evaluation of antibiotic resistance from bile culture, comparing malignant and benign obstructions in acute cholangitis patients.

Antibiotic Resistance *n*/Number of Antibiograms, %	Total (*n* = 266)	Malignant (*n* = 119)	Benign (*n* = 147)	Significance
Ampicillin/Sulbactam	21/67 (31.3%)	6/22 (27.2%)	15/45 (33.3%)	0.780
Piperacillin/Tazobactam	36/207 (17.3%)	18/91 (19.7%)	18/115 (15.6%)	0.464
Ciprofloxacin	45/225 (20.0%)	25/99 (25.2%)	20/125 (16.0%)	0.095
Levofloxacin	18/104 (17.3%)	6/37 (16.2%)	12/67 (17.9%)	0.999
Cefepime	41/196 (20.9%)	28/84 (33.3%)	13/111 (11.7%)	0.001
Ceftriaxone	11/73 (15.0%)	3/22 (13.6%)	8/51 (15.6%)	0.999
Ceftazidime	46/192 (23.9%)	30/82 (36.5%)	16/110 (14.5%)	0.001
Meropenem	17/194 (8.7%)	13/84 (15.4%)	4/110 (3.6%)	0.004
Imipenem	20/197 (10.1%)	17/84 (20.2%)	3/113 (2.6%)	<0.001

Data reported as *n* (%) and calculated using the chi-square test and Fisher’s exact test unless specified differently. The percentage was computed from the number of patients with an antibiogram.

**Table 7:** Evaluation of bacterial resistance to antibiotics from bile culture in patients with acute cholangitis.

Isolated Microorganisms from Bile Cultures No. of Patients (%)	*Escherichia coli* *n* = 92/192 (47.9%)		*Klebsiella* spp. *n* = 52/192 (27.1%)		*Pseudomonas* spp. *n* = 23/192 (11.9%)		*Enterococcus* spp. *n* = 41/192 (21.4%)	
	Malignant	Benign	Malignant	Benign	Malignant	Benign	Malignant	Benign
	34.8%	64.2%	48.1%	51.9%	52.2%	47.8%	51.2%	48.8%
	(32/92)	(60/92)	(25/52)	(27/52)	(12/23)	(11/23)	21/41	20/41
Ampicillin/Sulbactam	36.4%	46.7%	0%	33.3%	0%	0%	0%	0%
	(4/11)	(14/30)	(0/6)	(3/9)	(0/0)	(0/0)	(0/2)	(0/0)
Piperacillin/Tazobactam	9.4%	25.9%	20%	18.5%	33.3%	22.2%	0%	0%
	(3/32)	(15/58)	(5/25)	(5/27)	(4/12)	(2/9)	(0/0)	(0/0)
Ciprofloxacin	25%	19.2%	19%	11.5	25	10%	40%	50%
	(7/28)	(10/52)	(4/21)	(3/26)	(3/12)	(1/10)	(6/15)	(9/18)
Levofloxacin	0%	8%	0%	11.1%	20%	0.5%	26.7%	50%
	(0/7)	(2/25)	(0/2)	(1/9)	(1/5)	(0/4)	(4/15)	(9/18)
Cefepime	31%	16.7%	34.8%	0%	27.3%	20%	0%	0%
	(9/29)	(9/54)	(8/23)	(0/27)	(3/11)	(2/10)	(0/0)	(0/0)
Ceftriaxone	18.2%	17.2%	0%	22.2%	100%	0%	0%	0%
	(2/11)	(5/29)	(0/5)	(2/9)	(1/1)	(0/1)	(0/0)	(0/0)
Ceftazidime	33.3%	22.6%	34.8%	7.7%	20%	30%	0%	0%
	(10/30)	(12/53)	(8/23)	(2/26)	(2/10)	(3/10)	(0/0)	(0/0)
Meropenem	0%	0%	16.7%	3.8%	40%	22.2%	0%	0%
	(0/29)	(0/53)	(4/24)	(1/26)	(4/10)	(2/9)	(0/0)	(0/0)
Imipenem	0%	1.8%	17.4%	3.7%	60%	20%	0%	0%
	(0/30)	(1/55)	(4/23)	(1/27)	(6/10)	(2/10)	(0/0)	(0/0)

## Data Availability

Data are available on request.
